# Hyperbaric Oxygen Therapy Does Not Have a Negative Impact on Bone Signaling Pathways in Humans

**DOI:** 10.3390/healthcare9121714

**Published:** 2021-12-10

**Authors:** Zaida Salmón-González, Javier Anchuelo, Juan C. Borregán, Alvaro del Real, Carolina Sañudo, Maria Teresa García-Unzueta, José A. Riancho, Carmen Valero

**Affiliations:** 1 Department of Internal Medicine, Hospital Universitario Marqués de Valdecilla, IDIVAL, University of Cantabria, 39008 Santander, Spain; zaida.salmon@scsalud.es (Z.S.-G.); alvarodel.real92@gmail.com (A.d.R.); carolinasanudo@gmail.com (C.S.); jose.riancho@unican.es (J.A.R.); 2 Service of Radiation Oncology, Hospital Universitario Marqués de Valdecilla, 39008 Santander, Spain; javiertomas.anchuelo@scsalud.es; 3 Service of Intensive Care, Hospital Universitario Marqués de Valdecilla, 39008 Santander, Spain; juancarlos.rborregan@scsalud.es; 4 Service of Clinical Biochemistry, Hospital Universitario Marqués de Valdecilla, IDIVAL, 39008 Santander, Spain; mteresa.garciau@scsalud.es

**Keywords:** hyperbaric oxygen therapy, osteoprotegerin, RANKL, sclerostin, DKK1, HIF-1α

## Abstract

Introduction: Oxygen is emerging as an important factor in the local regulation of bone remodeling. Some preclinical data suggest that hyperoxia may have deleterious effects on bone cells. However, its clinical relevance is unclear. Hence, we studied the effect of hyperbaric oxygen therapy (HBOT) on serum biomarkers reflecting the status of the Wnt and receptor activator of NF-κB ligand (RANKL) pathways, two core pathways for bone homeostasis. Materials and methods: This was a prospective study of 20 patients undergoing HBOT (mean age 58 yrs., range 35–82 yrs.) because of complications of radiotherapy or chronic anal fissure. Patients were subjected to HBOT (100% oxygen; 2.4 atmospheres absolute for 90 min). The average number of HBOT sessions was 20 ± 5 (range 8–31). Serum hypoxia-inducible factor 1-α (HIF1-α), osteoprotegerin (OPG), RANKL, and the Wnt inhibitors sclerostin and dickkopf-1 (DKK1) were measured at baseline and after HBOT by using specific immunoassays. Results: HIF-1α in eight patients with measurable serum levels increased from 0.084 (0.098) ng/mL at baseline to 0.146 (0.130) ng/mL after HBOT (*p* = 0.028). However, HBOT did not induce any significant changes in the serum levels of OPG, RANKL, sclerostin or DKK1. This was independent of the patients’ diagnosis, either neoplasia or benign. Conclusion: Despite the potential concerns about hyperoxia, we found no evidence that HBOT has any detrimental effect on bone homeostasis.

## 1. Introduction 

Bone tissue is continuously remodeled by the concerted action of bone-resorbing osteoclasts and bone-forming osteoblasts. The Wnt and RANKL pathways are master regulators of bone remodeling [1]. Wnt ligands are expressed by a variety of skeletal and non-skeletal cell types. Within the bone microenvironment, they are critical factors promoting the proliferation and differentiation of mesenchymal stem cells into the bone-forming osteoblasts [2] The activity of the Wnt pathway within bone tissue is critically determined by the local levels of sclerostin and DKK1 [3,4]. These two soluble factors are secreted by osteocytes and other bone cells and prevent the activation of Wnt receptors by Wnt ligands [5]. The central role of these factors is revealed by the anabolic effect and increase in bone mass observed after therapy with neutralizing antibodies against sclerostin and DKK1, both in experimental models and in clinical practice [6].

Osteoclasts, the cells responsible for bone resorption, originate from hematopoietic precursors of the monocyte lineage. The binding of RANKL to RANK receptors present in osteoclast precursors is critical for their differentiation toward mature osteoclasts, as revealed by the profound inhibition of bone resorption induced by anti-RANKL antibodies, which are a well-established therapy for osteoporosis [7]. Osteoprotegerin (OPG) is an endogenous soluble decoy receptor for RANKL that prevents the binding of RANKL to its receptor RANK. Thus, the RANKL/OPG ratio in the bone microenvironment modulates osteoclast formation and consequently bone resorption.

Oxygen is critical for maintaining cell function, including that of bone cells [8]. In some experimental models, hypoxia stimulates the activity of bone-resorbing osteoclasts and inhibits the activity of bone-forming osteoblasts [9,10]. In contrast, hyperoxia may have opposite effects [11,12]. Hyperbaric oxygen therapy (HBOT) has anti-inflammatory effects [13], and it has been shown to be useful in the treatment of radiation-therapy-related complications (such as hemorrhagic cystitis or proctitis) and also in some bone disorders such as osteomyelitis [14], bisphosphonate-related osteonecrosis of the jaw [15], femoral head necrosis [16], and transient osteoporosis [17]. HBOT increases the partial pressure of oxygen in plasma and in tissues. However, the effects of HBOT on bone cells in vivo is unclear. HBOT could have either beneficial effects on bone, regulating the expression of hypoxia-inducible factor 1α (HIF-1α) [18], or detrimental effects related to oxidative damage [19]. To clarify this issue, we studied the effect of HBOT on serum levels of biomarkers of the Wnt and RANKL/OPG pathways. 

## 2. Materials and Methods

This was a prospective study of 20 patients subjected to HBOT. The mean age was 58 yrs. (range 35–82 yrs.), with 40% men and 60% women. Ten of them had a history of neoplasms, without bone metastases, and HBOT was indicated by the clinicians in charge due to complications of radiation therapy (proctitis 50%, cystitis 30%, and radionecrosis 20%; average total radiotherapy dose 50.7 Gy). Ten other patients without cancer underwent HBOT for chronic anal fissure that did not improve with conventional therapy.

HBOT was applied with a hyperbaric chamber (Galeazzi, Livorno, Italy), with 100% oxygen, at 2.4 atmospheres for 90 min while breathing through an oral-nasal mask, 5 times a week, with an average of 20 ± 5 sessions. The study protocol was approved by the institutional review board, and all patients provided written informed consent.

Baseline blood samples were obtained before starting the first HBOT session, and the second sample was extracted 30 min after the end of the last HBOT session. Serum aliquots were stored at −80 °C until analysis. Serum concentrations of HIF1-α, OPG, RANKL, sclerostin, and DKK1 were analyzed with immunoassays kits according to the manufacturers’ instructions. Both samples from each patient were analyzed within the same assay run. HIF-1α was measured by ELISA (Sigma-Aldrich, Saint Louis, MO, USA). The lower limit of detection was 61 pg/mL; the within-assay coefficient of variation (CV) was <10%. OPG was measured by ELISA (Sigma-Aldrich, Saint Louis, MO, USA). The lower limit of detection was 1 pg/mL; the intra-assay CV was <10%. RANKL was measured by ELISA (Biovendor, Brno, Czech Republic). The lower limit of detection was 0. 4 pmol/L; the intra-assay CV was <10%. DKK1 was measured by ELISA (Sigma-Aldrich). The lower limit of detection was 0.1 ng/mL; the intra-assay CV was <10%. Sclerostin was measured by ELISA (Tecnomedical, San Diego, CA, USA). Due to sample limitations, it was measured only in 12 patients. The lower limit of detection was 0.01 ng/mL; the intra-assay CV was <10%. C-reactive protein was quantified by an immunonephelometric assay (Behring Nephelometer Analyzer II, Behring Diagnostics, Marburg, Germany) using the ultrasensitive method. Analytical sensitivity was 0.03 mg/dL; the intra-assay CV was <3%. The normal reference range is 0.01–0.3 mg/dL.

### Statistical Analysis

Medians and interquartile ranges were used as the summary parameters. The Wilcoxon test for paired data was used to compare the baseline and post-HBOT serum biomarker levels using SPSS 15.0 software (IBM, Chicago, IL, USA). A *p*-value of <0.05 was considered statistically significant.

## 3. Results

The main clinical characteristics of the studied patients are listed in Table 1. The average number of HBOT sessions was 20 ± 5 (range 8–31), similar in both groups (23 ± 5 in neoplasms and 18 ± 4 in anal fissure).

**Figure 1:**
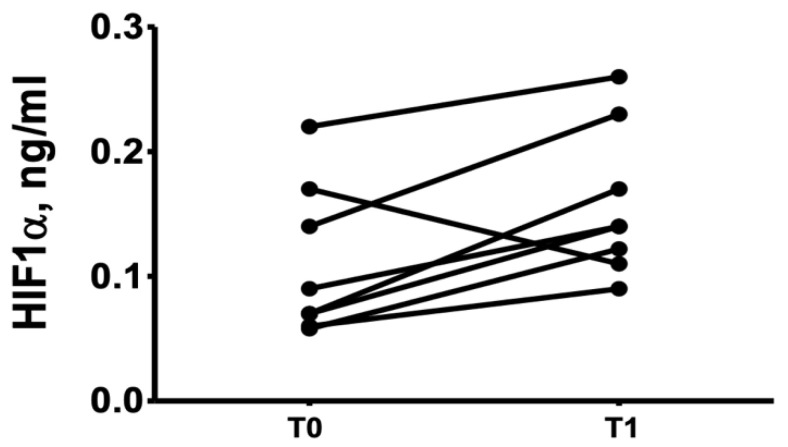
Levels of HIF-α before (T0) and after HBOT (T1).

HIF-1α serum levels were below the limit of detection in 12 patients. Among those with detectable levels, HIF-1α increased by 63% with HBOT, from 0. 084 (0.098) ng/mL at baseline to 0.146 (0.130) ng/mL after HBOT (*p* = 0.028) (Figure 1). 

No changes were found in the levels of C-reactive protein: 0.31 (0.62) mg/dL in T0 and 0.21 (0.90) mg/dL in T1 (*p* = 0.37) 

There were no significant changes after HBOT in serum OPG, RANKL, sclerostin, or DKK1 levels in comparison with baseline levels (Table 2).

No HBOT-induced differences in bone biomarkers were observed either when patients were stratified according to diagnosis (Table 3 and Table 4).

## 4. Discussion

The effects of oxygen on body homeostasis are mediated in part by HIFs. HIFs are heterodimeric proteins composed of a HIF-α and a HIF-β subunit. HIF-1α has an important role in the regulation of genes related to bone metabolism [21]. In normoxia (>5%), prolyl hydroxylase domain (Phd) proteins hydroxylate proline residues in HIF-α subunits. In hypoxia, HIF-α hydroxylation by Phd is prevented, and HIF-α accumulates, associates with HIF-β, and induces the expression of target genes, increasing osteoclast activity. The effect on osteoblast activity is more complex, because it may be reduced by HIFs, but may be indirectly stimulated by the induction of vascular endothelial growth factor (VEGF) [22]. The expression of HIF in hyperoxia-associated situations seems to be rather complex, too. A study in vivo with human peripheral blood mononuclear cells showed that the return to normoxia after 1 hour of exposure with mild or high oxygen (30% and 100% O_2_, respectively) resulted in a hypoxia-like response, characterized by increased HIF-1α. However, the exposure to high oxygen concentrations did not result in changes in HIF-1α [19] but to an oxidative stress response that could be detrimental to bone due to increased reactive oxygen species (ROS) and oxidative damage [23,24,25]. 

In this study, we found increased HIF-1α levels after HBOT, but this did not seem to impact the major drivers of osteoblast and osteoclast differentiation. The OPG/RANK/RANKL system regulates osteoclastogenesis. OPG, the soluble decoy receptor for RANKL, inhibits RANKL binding to RANK and prevents bone resorption [26]. Hypoxia and hyperoxia could modulate the RANKL/OPG ratio [27,28]. However, we did not find any HBOT-induced changes in serum OPG or RANKL levels. These results are consistent in part with those of a previous study of patients with femoral head necrosis, which found that OPG increases with HBOT, whereas RANKL does not change [29]. However, it is hard to know whether OPG changes were due to oxygen therapy itself or to the potential modifications of the bone lesion. 

The Wnt pathway is a master regulator of osteoblastogenesis [30]. Oxygen levels influence the functioning of this signaling pathway in experimental models [31]. Sclerostin, the product of the SOST gene, is a secreted inhibitor of Wnt signaling that is produced by osteocytes and tends to decrease bone formation [32]. Hypoxia decreases SOST expression and consequently increases Wnt signaling in osteoblasts [33]. Thus, although these effects have not been confirmed in all studies [34,35], hypoxia could result in increased bone formation, whereas hyperoxia could decrease it. However, the results in different experimental systems are rather contradictory. In fact, it has been reported that HBOT increases the osteogenic differentiation of mesenchymal stem cells in vitro [12], whereas in an animal model, HBOT appeared to protect from the osteoporosis induced by hind limb unloading through decreased SOST expression [36]. DKK1 is another important modulator of the Wnt pathway [37]. Hypoxia seems to modulate the expression of DKK1 [38], but the effects of hyperoxia have not been studied. In our study, we did not find any evidence of increased levels of sclerostin or DKK1 in patients who were exposed to HBOT. Overall, these results are in line with the absence of significant changes in bone turnover markers in patients exposed to HBOT, as well as with the limited effects of HBOT on bone samples ex vivo [35]. 

HBOT may have anti-inflammatory effects [39,40], which may secondarily influence bone metabolism [41]. The lack of effects of HBOT on CRP levels in this study must be interpreted, taking into consideration the fact that our patients already had low CRP levels at baseline, consistent with the absence of marked active inflammation. 

Our study had several limitations, including a moderate sample size, which resulted in a relatively high type II error and limited the statistical power. We also consider a limitation the absence of direct measurements within the bone microenvironment medium. HIF-1α is a rather labile compound, which may have contributed to the fact that it was unmeasurable in some patients. In addition, since we did not measure other mediators of the response to oxygen levels (i.e., Phd, HIF-2), we cannot exclude other effects of HBOT. In addition, importantly, our results cannot be necessarily generalized to other conditions and HBOT schedules. Nevertheless, our results about serum biomarkers are reassuring. We did not find any evidence of a deleterious effect of HBOT, as applied in this study, on the activity of skeletal cells.

## Figures and Tables

**Table 1:** Main clinical features of the study population.

Age (yrs.), Mean (SD)	58 (15)
Sex male, *n* (%)	8 (40%)
Physical activity, *n* (%)	
-Sedentary (<2 h/week)	2 (10%)
-Moderate (2–4 h/week)	14 (70%)
-Vigorous (>4 h/week)	4 (20%)
Alcohol consumption, *n* (%)	4 (20%)
Smoker habit, *n* (%)	2 (10%)
Charlson comorbidity index, *n* (%)	
-0	5 (25%)
-1–2	11 (55%)
-3–4	3 (15%)
->4	1 (5%)
HBOT indication, *n* (%)	
-Anal fissure	10 (50%)
-Proctitis	5 (25%)
-Cystitis	2 (10%)
-Radionecrosis	3 (15%)
Tumor type, *n* (%)	
-Adenocarcinoma rectum/colon	3 (30%)
-Carcinoma head and neck	3 (30%)
-Carcinoma prostate	2 (20%)
-Adenocarcinoma gynecological	2 (20%)

Charlson comorbidity index [20].

**Table 2:** Levels of bone biomarkers before and after HBOT.

Bone Biomarkers	Baseline (*n* = 20)	After HBOT (*n* = 20)	*p*-Value
OPG pg/mL	154 (62)	165 (64)	0.73
RANKL pmol/L	352 (249)	334 (228)	0.50
Sclerostin * ng/mL	0.87 (1.38)	0.73 (0.67)	0.11
DKK1 ng/ml	2.88 (6.22)	3.15 (8.01)	0.39

Median (and interquartile range). OPG: osteoprotegerin; RANKL: receptor activator for nuclear factor kappa B ligand; DKK1: dickkopf-1. * Sclerostin levels were measured only in 12 patients.

**Table 3:** Bone biomarkers in patients with neoplasms.

Bone Biomarkers	Baseline (*n* = 10)	After HBOT (*n* = 10)	*p*-Value
OPG pg/mL	155 (78)	173 (97)	0.24
RANKL pmol/L	357 (202)	343 (262)	0.50
Sclerostin * ng/mL	1.16 (1.18)	0.65 (1.28)	0.18
DKK1 ng/mL	1.62 (3.03)	1.81 (2.94)	0.87

Median (and interquartile range). OPG: osteoprotegerin; RANKL: receptor activator for nuclear factor kappa B ligand; DKK1: dickkopf-1. * Sclerostin levels were measured only in 6 patients.

**Table 4:** Bone biomarkers in patients with anal fissure.

Bone Biomarkers	Baseline (*n* = 10)	After HBOT (*n* = 10)	*p*-Value
OPG pg/mL	148 (79)	159 (82)	0.12
RANKL pmol/L	332 (431)	323 (252)	0.47
Sclerostin * ng/mL	0.57 (1.58)	0.83 (1.02)	0.11
DKK1 ng/mL	3.35 (8.98)	3.44 (8.12)	0.33

Median (and interquartile range). OPG: osteoprotegerin; RANKL: receptor activator for nuclear factor kappa B ligand; DKK1: dickkopf-1. * Sclerostin levels were measured only in 6 patients.

## Data Availability

The data presented in this study is private.
